# Simulation-Based Optimization over Discrete Spaces Using Projection to Continuous Latent Spaces

**DOI:** 10.1021/acs.iecr.5c04315

**Published:** 2026-02-13

**Authors:** Gabriel Hernández-Morales, Brenda Cansino-Loeza, Arturo Jiménez-Gutiérrez, Victor M. Zavala

**Affiliations:** † Department of Chemical and Biological Engineering, 5228 University of Wisconsin - Madison, 1415 Engineering Dr, Madison, Wisconsin 53706, United States; ‡ Department of Chemical Engineering, Tecnologico Nacional de Mexico - Instituto Tecnologico de Celaya, 38010 Celaya, Guanajuato, Mexico

## Abstract

Simulation-based optimization of complex systems over discrete decision spaces is a challenging computational problem. Specifically, discrete decision spaces lead to a combinatorial explosion of possible alternatives, making it computationally prohibitive to perform simulations for all possible combinations. In this work, we present a new approach to handle these issues by transforming/projecting the discrete decision space into a continuous latent space using a probabilistic model known as Variational AutoEncoders. The transformation of the decision space facilitates the implementation of Bayesian optimization (BO), which is an efficient approach that strategically navigates the space to reduce the number of expensive simulations. Here, the key observation is that points in the latent space correspond to decisions in the original mixed-discrete space, but the latent space is much easier to navigate using BO. We illustrate the benefits of our approach through a couple of case studies that aim to design complex distillation systems: the recovery of caprylic acid from water by liquid–liquid extraction and the separation of an azeotropic mixture using a thermally couple column known as an extractive dividing wall column.

## Graphical abstract

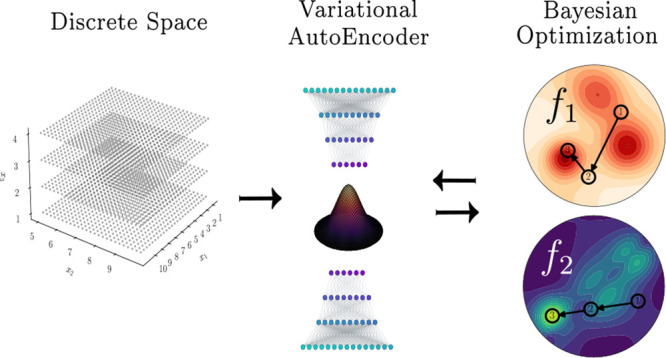

## 1 Introduction

The design of complex systems, such as chemical processes, often involves a search space over discrete variables (e.g., topology of the chemical process, solvent selection, or equipment selection). The presence of discrete decisions complicates the use of optimization techniques, particularly when the system model is a black-box simulator (there is no explicit access to algebraic equations). This challenge has motivated the development of black-box optimization strategies, which rely on observed input—output data obtained from the simulator.^1,2^ Among these approaches, genetic algorithms (GAs) and other evolutionary algorithms have been widely used and applied to various processes with different levels of complexity.^3–6^ Evolutionary algorithms are meta-heuristic approaches that can handle discrete design spaces, but they might require a large number of simulations and there are no clear guidelines on when to stop the search. Similar limitations are often encountered with other meta-heuristic approaches.^7^

Bayesian Optimization (BO) has emerged as a powerful black-box optimization method due to its ability to efficiently explore design spaces using probabilistic surrogate models (usually a Gaussian Process-GP) that are trained on limited data, making it useful when simulations or experiments are costly. A key aspect of BO is that it leverages uncertainty information on the surrogate model to help explore the design space.^8,9^ The application of BO to the design of chemical processes, such as the design of separation systems, has shown that this approach is effective at reducing the number of simulations. However, existing studies have focused on decision spaces that only involve continuous decisions, such as distillate-to-feed and reflux ratios.^10,11^ In order to handle discrete variables in BO, some studies have used rounding strategies;^12^ this approach, however, can lead the algorithm to get trapped in local optima or can lead to infeasible solutions. For example, the work reported in Luong et al.^13^ proposed an approach tailored for discrete search spaces that avoids redundant sampling and local optima by optimizing the exploration-exploitation factor and the scaling length of the covariance function. While this method improves performance in purely discrete spaces, the proposed modifications are ad-hoc. The work in Wan et al.^14^ proposed a BO method for categorical and mixed-discrete domains, combining customized kernels with a local trust region strategy for high-dimensional optimization. However, it is in general difficult to fit a smooth surrogate model over a discrete space.

Researchers in the molecular design community have proposed to transform the molecular design space (which is inherently discrete) into a continuous space by using *Variational AutoEncoders* (VAE).^15^ VAEs aim to identify latent representations of input data; this is done by training a neural network (known as an encoder) that compresses the input into a lower-dimensional space and by simultaneously training another neural network (known as a decoder) that aims to reconstruct the original input data from the latent representation. This approach has been widely used to navigate the design space of molecules and proteins. Building on this concept, the work in Stanton et al.^16^ introduced LaMBO, a BO strategy that employs a low-dimensional latent space and an autoregressive model to optimize small molecules and protein sequences. This methodology was extended with LaMBO-2 in Gruver et al.,^17^ which replaces Gaussian process (GP) models with ensemble methods, maintaining the focus on discrete spaces. Other works have also explored the use of BO over discrete inputs, using string-based kernels or exploiting the structure of the latent space in molecular applications.^18–20^ More recently, Michael et al.^21^ proposed CoRel, an approach for protein sequence optimization that reformulates the discrete BO problem into a continuous one via a VAE latent space. This methodology enables the direct definition of a GP model over probability distributions, utilizing a new kernel based on the weighted Hellinger distance. This formulation not only incorporates prior domain knowledge but also proves to be effective in low-budget optimization scenarios, such as protein design, where data are scarce and experimental evaluations are expensive. The use of autoencoders to transform complex spaces into tractable ones (e.g., from nonlinear to linear) has also found many uses in the development of dynamical models; specifically, diverse studies have reported on the ability of using autoencoders to help discover linear latent spaces under which it is much easier to develop dynamical models and control strategies.^22^

In this work, we aim to investigate whether discrete spaces encountered in simulation-based process design can be represented as continuous spaces using VAEs. In contrast to these biology-focused methods, which tailor generative models to sequence optimization, our work uses a conventional VAE as a direct encoder—decoder mapping from discrete distillation-column design variables to a continuous latent space. We use a BO algorithm to navigate the continuous latent space, to guide the strategic collection of simulation data over the design space, and to identify optimal designs. We apply our framework to a couple of case studies arising in the design of complex separation systems. Thus, the contribution of this work lies in embedding discrete process design decisions into a continuous latent space suitable for BO. Our results provide interesting insights into the ability of using VAEs to transform complex decision spaces and to facilitate their navigation. Specifically, we show that the proposed approach can help identify designs of high quality with a few simulations. While our results are computational and empirical in nature, we believe that the use of VAEs offer an interesting and practical alternative to help navigate complex decision spaces arising in optimization. All data and code needed to reproduce our results can be found at https://github.com/zavalab/ML/tree/master/bayesianvae.

## 2 Projection of Discrete into Continuous Spaces

A variational autoencoder (VAE) is a machine learning model that aims to learn a latent space from which original data can be reconstructed with minimal information loss.^15^ A VAE is a compression–decompression system that operates as follows: the *encoder* takes points that live in an input discrete space x∈X and compresses them using a sequence of nonlinear transformations (conducted using a neural network) into points that live in a continuous latent space z∈Z . The *decoder* uses a sequence of nonlinear transformations (conducted using another neural network) to reconstruct the original points from points in the latent space in a way that it minimizes a measure of reconstruction error. The parameters of the VAE model are trained using available data (in an unsupervised manner). For simplicity in the discussion, we assume that the input space X is completely discrete (it does not include continuous variables); any continuous variables can be processed via discretization. In practice (e.g., in experiments), one often implements continuous variables in discrete values. The concepts discussed can be generalized to account for input spaces that are mixed-discrete.

Unlike a standard autoencoder, which builds a latent space that is unstructured,^19^ the VAE architecture is unique in that it induces a probabilistic structure into the latent space. Specifically, each data point in the original space is encoded not as a single point but as a probability distribution (typically a Gaussian distribution). During training, samples from this distribution are drawn to force the decoder to reconstruct the original input from noisy/perturbed latent vectors. This probabilistic step ensures that the latent space is smooth and continuous, meaning that nearby points in the latent space correspond to similar configurations in the original space. Smoothness of the latent space makes this easier to explore, interpolate over, and optimize. For example, in a process design, instead of working directly with a decision space that handles discrete variables (e.g., number of stages and the location of the feed stage), the VAE transforms these into a continuous latent representation. The smoothness of the latent space facilitates the development of surrogate models, such as GP models.

A schematic of a typical VAE architecture is presented in Figure 1. Data for each input variable is transformed into a continuous vector **e** using an embedding layer. Embedding enables learning dense, low-dimensional representations that are adjusted during the training process of the encoder network to capture semantic relationships between the embedded items, which enables the conversion of discrete input data into continuous, learnable representations, which are essential for many machine learning models. In this context, the variables of this layer are learnable weights initialized from a N(0,1) distribution and optimized via backpropagation. The embedded representations **e** are combined into a single vector **h** through concatenation. Subsequently, **h** passes through a multilayer perceptron layer (MLP) to decrease the size until a given desired latent space dimension is reached. The embedding layer and the MLP are denoted as the encoder and are represented by the mapping ϕ. The last layers of the encoder are used to perform a reparameterization to add stochasticity into the latent space **z**:^23^

$$z=\mu +\epsilon \cdot \exp\left(\frac{1}{2}\;\log\sigma^{2}\right),\epsilon ∼N\left(0,I\right)$$ 1

where **ϵ** is standard Gaussian noise with mean zero and variance of one, **μ** is the mean vector which are mean values for the latent variables and **σ** the standard deviation vector (that implicitly captures the range of possible values for the latent variables). Symbol **I** is the identity matrix, indicating that all components of **ϵ** are independent standard normal variables. The probabilistic reparameterization enables the use of gradient-based optimization. The decoder (denoted as the inverse mapping ϕ^–1^), takes a point in the latent space **z** and predicts logits values **y**, which are used to predict probabilities using the softmax function S(·). This framework allows the reconstruction of the original input by categorical probabilities or by discrete class predictions.

**Figure 1:**
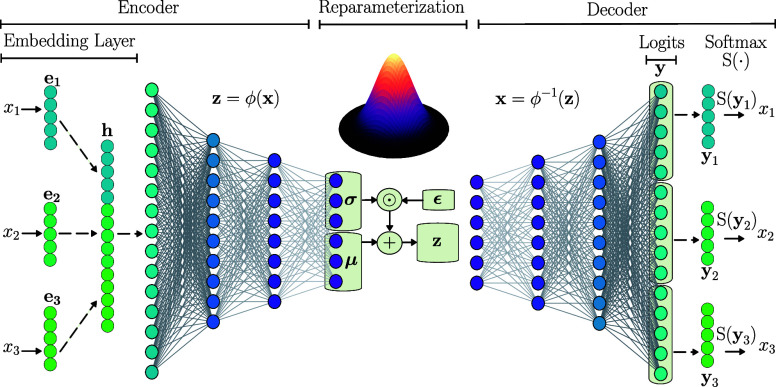
VAE architecture designed for a set of 3 discrete inputs $x=\left[x_{1},x_{2},x_{3}\right]\in X$ ; each discrete input is mapped into a continuous representation **e** through an embedding layer. The embeddings are concatenated into a vector **h** and processed by the encoder. The encoder produces the mean vector **μ** and the standard deviation vector **σ**, which are used to generate the latent space Z . The decoder maps points in the latent space z∈Z back into the original space by producing logits **y** for each input variable; a softmax function is used to obtain probability distributions over the possible values of *x*_1_, *x*_2_, and *x*_3_ to reconstruct the original discrete space (analogous to a classification model).

The VAE is trained (the parameters of the encoder and decoder networks are learned) to minimize a composite loss function that captures the cross-entropy loss $L_{\mathrm{rec}}$ and the Kullback–Leibler divergence $L_{\mathrm{KL}-D}$ loss. The cross entropy $L_{\mathrm{rec}}$ captures individual entropies for each discrete variable and measures the ability to reconstruct the original space from the latent space (which is a measure of the reconstruction error):

2 $$L_{\mathrm{rec}}=-\overset{N}{\underset{n=1}{\sum }}\overset{C}{\underset{c=1}{\sum }}\;\log\left(\frac{e^{y_{n,x_{n}}}}{\overset{C}{\underset{i=1}{\sum }}e^{y_{n,i}}}\right)$$

Here, *C* is the number of possible classes/values for each discrete variable, and *N* the total number of input discrete variables. Symbol *x*_*n*_ denotes the index value used to measure the distance between the true class (discrete value) and the predicted class (reconstructed value). To estimate the $L_{\mathrm{rec}}$ loss, the logit values **y** predicted by the decoder are used to compute it.

The Kullback–Leibler divergence $L_{\mathrm{KL}-D}$ measures how far the learned latent distribution $q\left(z|x\right)∼N\left(\mu ,\sigma^{2}\right)$ is from the target standard normal distribution p(z)∼N(0,I) :

3 $$L_{\mathrm{KL}-D}=\frac{1}{2}\overset{D}{\underset{j=1}{\sum }}\left(\exp\left(\log\sigma_{j}^{2}\right)+\mu_{j}^{2}-\log\sigma_{j}^{2}-1\right)$$

We note that the Kullback–Leibler divergence acts as a regularization term that aims to induce a structure on the latent space.

The composite loss function is given by

4 $$L=L_{\mathrm{rec}}+\beta \cdot L_{\mathrm{KL}-D}$$

where $\beta \in R_{+}$ is a weighting parameter that trades-off the loss functions. Thus, the number of neurons in each hidden layer of the encoder and decoder, the slope of the activation function (LeakyReLU), β, the learning rate, the dimensionality of **z**, and the embedding size are considered hyperparameters to be optimized. Evaluating the final model requires fine-tuning the architecture of the VAE to reduce the global loss function L . To achieve this, a 5-fold cross-validation is performed after 250 epochs. Once the optimal hyperparameters have been identified, the final model is trained for 1000 epochs, which further improves the overall performance of the model.

In summary, the use of a VAE offers a couple of important benefits: (i) it will represent discrete variables in a smooth and continuous space and (ii) it will provide a bridge between the original problem formulation with advanced optimization tools (such as BO), which benefit from smooth search spaces.

To illustrate the applicability of the VAE, we consider a simple problem of designing a reactor with the goal of maximizing the production of component *C*, which is produced simultaneously through a couple of competing routes, a consecutive pathway $\left(A\to^{k_{1}}B\to^{k_{2}}C\right)$ (referred to as Pathway 1) and a parallel direct pathway $\left(A\to^{k_{3}}C\to^{k_{4}}D\right)$ (referred to as Pathway 2). The kinetics of the reactor system are given by a set of elementary reactions, each characterized by a rate constant dependent on temperature *k*_*i*_(*T*) that follows the Arrhenius equation, $k_{i}\left(T\right)=A_{i}\;\exp\left(-\frac{E_{i}}{\mathrm{RT}}\right)$ . We note from Pathway 2 that accelerating the formation of *C* simultaneously enhances its undesired degradation to *D*. Each pathway has its own maximum production, which depends on both temperature and time. The goal is thus to determine the operating conditions that balance these competing effects and maximize the production of specie *C*, considering both pathways. The selection of the reaction pathway is a discrete design decision; this could be manipulated, for instance, by changing the catalyst. The continuous decision variables include reaction time and temperature, which are discretized from their continuous spaces.

Within the discrete decision space, we evaluate the production of component *C* along each pathway, and we can visualize the production of *C* across the entire design space in Figure 2, where each point on this grid corresponds to a combination of temperature, reaction time, and pathway. This visualization highlights the presence of a couple of local productivity maxima. The discrete space is projected to the continuous latent space Z . By learning the encoder ϕ and decoder ϕ^–1^ mappings, a low-dimensional latent representation of the design space that captures its essential features is obtained; in addition, we obtain a mapping to reconstruct the original variables from this representation. This approach will allow the exploration of feasible solutions in the latent space while ensuring valid operating choices for temperature, time, and pathways.

**Figure 2:**
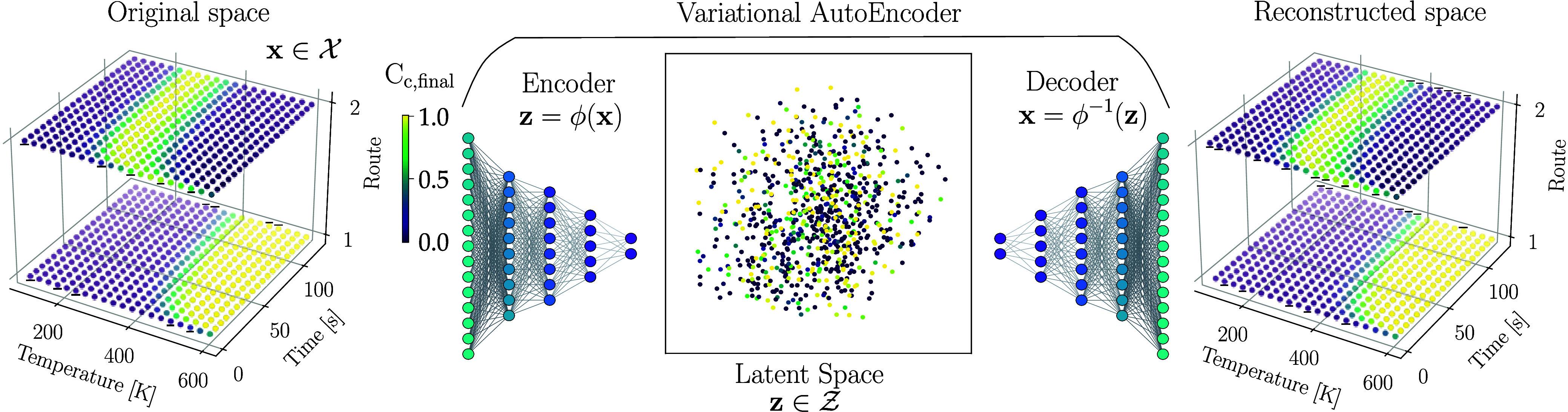
Schematic representation of the original discrete space X mapped through the decoder ϕ to obtain the latent continuous space Z from which we can reconstruct the original space using the decoder ϕ^–1^. Dots are colored based on the final concentration of C (C_*c*_) at those operating conditions.

For a suitable VAE architecture, one expect that the reconstruct of the original space with small reconstruction errors. A key challenge in enabling this is to find a suitable dimension of the latent space that minimizes the reconstruction loss. In Figure 3, we illustrate the influence of the dimension of the latent space on the loss function trained after 500 epochs. We note that the total (composite) loss stabilizes at a latent space dimension of eight. Here, we also present the profiles of each loss component after 500 epochs of VAE training, for illustrative purposes. Once the VAE can successfully reconstruct the original space from a latent space, we can proceed to use the latent space to search for optimal operating conditions with BO. Therefore, the optimal dimensionality of **z** is explored between six and eight. Lower-dimensional values will imply significant reconstruction errors, thereby resulting in poor performance for BO. Conversely, increasing the latent dimensionality beyond eight does not result in meaningful improvements in the VAE loss function and introduces unnecessarily high-dimensional search spaces, thereby reducing the efficiency of BO. In the next section, we will discuss in more detail how this can be done systematically using BO.

**Figure 3:**
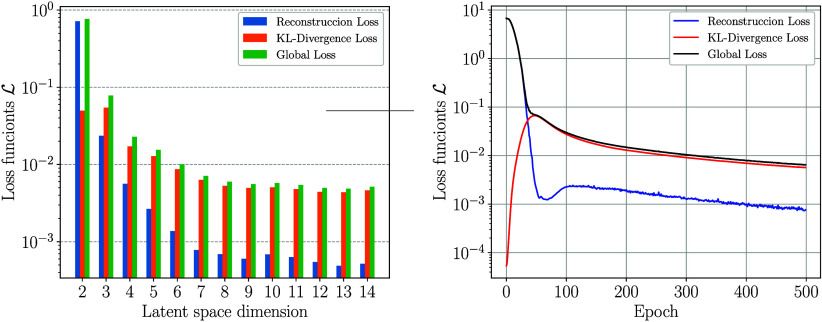
Impact of the latent space dimension on the composite loss function L and its components, the reconstruction term Lrec and the KL-divergence term $L_{\mathrm{KL}-D}$ for the illustrative reactor design example (left). Progression of the loss functions during the training of a VAE with a latent dimension of eight for the same reactor problem (right).

## 3 Bayesian Optimization (BO) in VAE Latent Space

Our goal is to solve the optimization problem:

5 $$\underset{x\in X}{\min}f\left(x\right)$$

The key challenge is that the evaluation of the objective (cost) function *f*(**x**) at a point x∈X requires a potentially expensive simulation. By transforming the original discrete design space X into a continuous space Z using a VAE, we can pose the optimization problem as

6 $$\underset{z\in Z}{\min}f\left(\phi^{-1}\left(z\right)\right)$$

By changing the search space, we now aim to navigate the latent space so that the objective function is minimized; every point in the latent space is converted into a point in the discrete space. To navigate through the latent space, we will use a BO approach; this will build a surrogate model *m*(**z**) that aims to approximate the composite mapping *f*(ϕ^–1^(**z**)) at a given point in the latent space z∈Z . With the use of the surrogate model, the BO approach navigates through the original design space implicitly by navigating through the latent space.

The combined BO+VAE framework is sketched in Figure 4. At iteration l , we have a set of data points $x^{l}\in X$ with their corresponding costs $f\left(x^{l}\right)$ . The input data points are encoded using ϕ to obtain the latent points $z^{l}\in Z$ and associated cost values $f\left(\phi^{-1}\left(z^{l}\right)\right)$ . We build a probabilistic surrogate model $m^{l}\left(z\right)$ to approximate the composite mapping *f*(ϕ^–1^(**z**)). The surrogate $m^{l}\left(z\right)$ is trained using the available cumulative database $D^{l}={\left\{z^{k},f\left(\phi^{-1}\left(z^{k}\right)\right)\right\}}_{k=1}^{l}$ . A Gaussian Process model ( GP ) is used as a surrogate model, which is a probabilistic model that provides a mean prediction $\mu_{f}^{l}\left(z\right)$ of the cost function and the uncertainty $\sigma_{f}^{l}\left(z\right)$ of the prediction.

**Figure 4:**
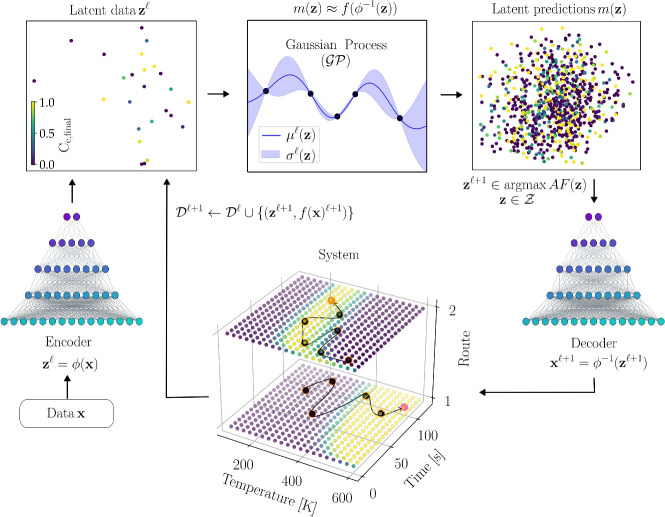
Illustration of integrated BO+VAE framework. Data **x** is mapped to **z** using ϕ. A surrogate model over the latent space *m*(**z**) is built and iteratively selects promising points in the latent space $z^{l+1}$ by optimizing an acquisition function (*AF*). Latent samples $z^{l+1}$ are decoded to obtain a simulation at the new point $f\left(\phi^{-1}\left(z^{l+1}\right)\right)$ . The surrogate model is refined by updating the data set $D^{l}$ .

We note that the BO+VAE framework includes a couple of approximation errors: the VAE reconstruction error (obtained by mapping from the latent to the original space) and the error of the surrogate model. An important observation is that, even if there are reconstruction errors, the refinement of the GP model should be able to correct for any discrepancies. However, we expect that the reconstruction error needs to be sufficiently small to ensure that points in the latent space are true reflections of points in the original space to have a consistent search.

BO selects a new decision by optimizing an acquisition function (*AF*) that balances exploration and exploitation. Exploration is driven mainly by the uncertainty, $\sigma_{f}^{l}\left(z\right)$ where new regions not explored are prioritized; exploitation focuses on the mean cost $\mu_{f}^{l}\left(z\right)$ predicted to refine the solution in a specific design space. To balance exploration and exploitation, we use the Expected Improvement (*EI*) as the acquisition function. The pseudocode for the standard Bayesian optimization algorithm, enhanced with the VAE for single-objective problems is described in Algorithm 1. We use $D^{\kappa }$ to denote the data set used to train the VAE; this data set is distinct from the data set $D^{l}$ used to initialize BO. $D^{\kappa }$ is composed of discrete feasible configurations sampled, and the amount of data required to cover the entire design space is crucial to perform BO. If the VAE is trained on scarce data, it is more likely to fail to converge to the true Pareto front.
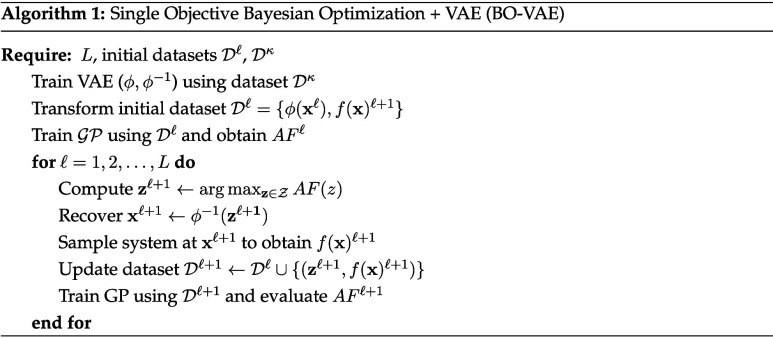


We are often interested in optimizing multiple objective functions to assess trade-offs. Here, we focus on bicriteria problems of the form:

7 $$\underset{z\in Z}{\min}\left(f_{1}\left(\phi^{-1}\left(z\right)\right),f_{2}\left(\phi^{-1}\left(z\right)\right)\right)$$

In this setting, the goal of BO is to discover the so-called Pareto frontier (set of nondominated points); to do so, we use a simple procedure in which we build a GP model for each cost function and use the predictive means $\mu_{f}^{l}\left(z\right)$ to identify the predicted nondominated points and thus predict the location of the Pareto front. At each point on the predicted Pareto frontier, we use the uncertainty information $\sigma_{f}^{l}\left(z\right)$ to determine the locations on the front that have the highest uncertainty. The next iteration $z^{l+1}$ is selected by identifying the point on the Pareto front with the highest uncertainty; as we will illustrate below, this simple data acquisition approach can discover the Pareto frontier with few simulations. The proposed approach is presented in Algorithm 2 for multiple objective functions and is referred to as MOBO-VAE.

The multiobjective framework was benchmarked against the Nondominated Sorting Genetic Algorithm (NSGA-II) proposed by Deb et al.^24^ This algorithm has the advantage of being simple and efficient at generating widely distributed solutions along the Pareto front. The best solutions in each iteration are identified using the crowding distance, which are then used in the next step to generate new points for evaluation. The primary hyperparameters that affect the performance of the algorithm are the starting number of points evaluated (population), the number of points tested in the following iteration (number of offspring), and the total number of iterations (generations). These hyperparameters are fixed according to the same parameters used for the multiobjective problem in each case study, ensuring a consistent benchmarking basis. Mutation and crossover probabilities are set to 0.9 to enable broad exploration of the search space, thereby enhancing overall performance. The NSGA-II approach is implemented in this work using the Pymoo package.^25^ 
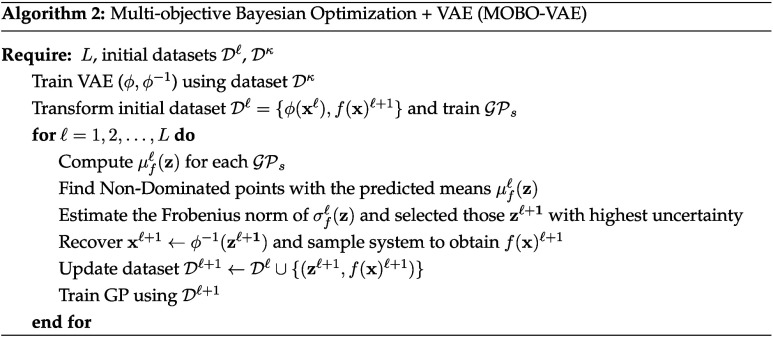


## 4 Case Studies

To assess the effectiveness of the proposed framework, we built a couple of detailed case studies that aim to design complex separation systems; the first system conducts liquid–liquid extraction of caprylic acid from water and the second system involves the separation of an azeotropic mixture by a dividing wall column. We implemented detailed simulation models for these systems using AspenPlus; our goal is to identify optimal designs for such systems by using as few expensive simulations as possible.

### 4.1 Liquid–Liquid Extraction of Caprylic Acid from Water

Caprylic acid (CA), a medium-chain fatty acid, is a platform chemical that has garnered significant attention due to its applications in the food and pharmaceutical industry.^26,27^ This component is mainly obtained through chain elongation of syngas during anaerobic fermentation. A challenge of this process is that fermentation yields highly diluted systems from which CA needs to be recovered; here, we design a liquid–liquid extraction system to address such challenge.

In Yuan et al.,^28^ an analysis was conducted to identify potential solvents (entrainers) that can be used to capture CA with high selectivity. Among the eight solvents tested, they found that oleyl alcohol, 1-decanol, 1-octanol, and *n*-butyl acetate have high selectivity. A schematic of the process is shown in Figure 5. A bioreactor where the anaerobic fermentation takes place is followed by a centrifuge operation that splits biomass, leaving a mixture of CA and water at 298.15 K with a total molar flow rate of 1210.04 kmol/h and 0.04 kmol/h for CA and water, respectively. Liquid–liquid extraction is performed using a 10-stage column, and this task can be conducted with either of the four solvents mentioned above. The remaining water is separated, and the final step consists of a distillation column where the distillate rate or bottom rate are fixed to 0.042 kmol/h (the value depends on the solvent selected). All units involved are considered assumed to operate at atmospheric pressure.

**Figure 5:**
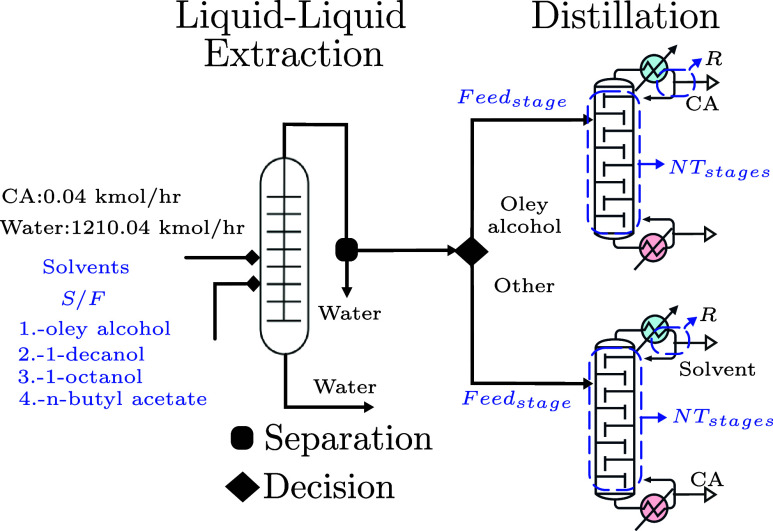
Process for the recovery of caprylic acid using liquid–liquid extraction. Blue text indicates all the design variables that are optimized.

The design aims to identify optimal solvents and configurations for the column that maximizes the molar fraction of CA (*x*_*CA*_), which is a metric of *separation efficiency*. The second design objective is to minimize the reboiler duty of the column (*Q*_*reb*_), which is a metric of *energy efficiency*. Let d∈D denote the *design decision variables* considered in Table 1 that rely on a mixed-design space.

$$\begin{array}{cc} \underset{d\in D}{\min} & \left(Q_{reb}\left(d\right),-x_{CA}\left(d\right)\right)\\ s.t. & g\left(d\right)\leq 0,\\ & h\left(d\right)=0, \end{array}$$ 8

where **h**(·) = **0** represents the column model equations (e.g., MESH/VLE/energy balances) and **g**(·) ≤ **0** represents operational and design constraints (e.g., feasibility bounds). The solution is the set of Pareto-optimal designs that balance product purity and energy demand. Thermodynamic data used are reported in the Supporting Information (SI). The design variables with their corresponding bounds are given in Table 1. The total amount of data generated from this information to train the VAE ( $D^{\kappa }$ ) was 20,000 points, during which constraints were explicitly imposed. The final hyperparameters (Table S1) and the performance profiles obtained after optimization are provied in the SI (Figure S1a).

**Table 1:** Design Variables of Caprylic Acid Recovery

**Design variables**	**Units**	**Range**	**Domain**
Solvent Selection (*S*)	[-]	{0, 1, 2, 3}^a^	Categorical
Reflux Ratio (*R*)	[-]	(0.1 – 3.0)	Continuous
Solvent Flow rate (*F*_*S*_)	kmol/h	(1.5 – 10.0)	Continuous
Feed Stage (*NF*_*stage*_)	[-]	{1 – 60}	Discrete
Total Number of Stages (*NT*_*stages*_)	[-]	{20 – 60}	Discrete

a Oleyl alcohol, 1-octanol, 1-decanol, and *n*-butyl acetate are encoded using categorical values of 0, 1, 2, and 3, respectively.

Figure 6a visualizes the latent space Z in 2 dimensions; this visualization was obtained using t-distributed Stochastic Neighbor Embedding (t-SNE). Here, we highlight the points in the latent space associated with different solvents, feed positions, and number of stages. It is interesting to observe that the VAE induces some mixing of the discrete design variables. In Figures 6b and 6c, one can observe the latent space highlighting locations, different purity and reboiler duty (the objectives). One can see that the VAE generates a latent space that is ellipsoidal, highlighting the fact that the latent space is structured as a Gaussian random variable. Also, there are diverse locations in the latent space (corresponding to different designs) that achieve high purity and low reboiler duty. These visualizations of the latent space leveraged the use of the GP model to predict the objectives in different regions of the search space.

**Figure 6:**
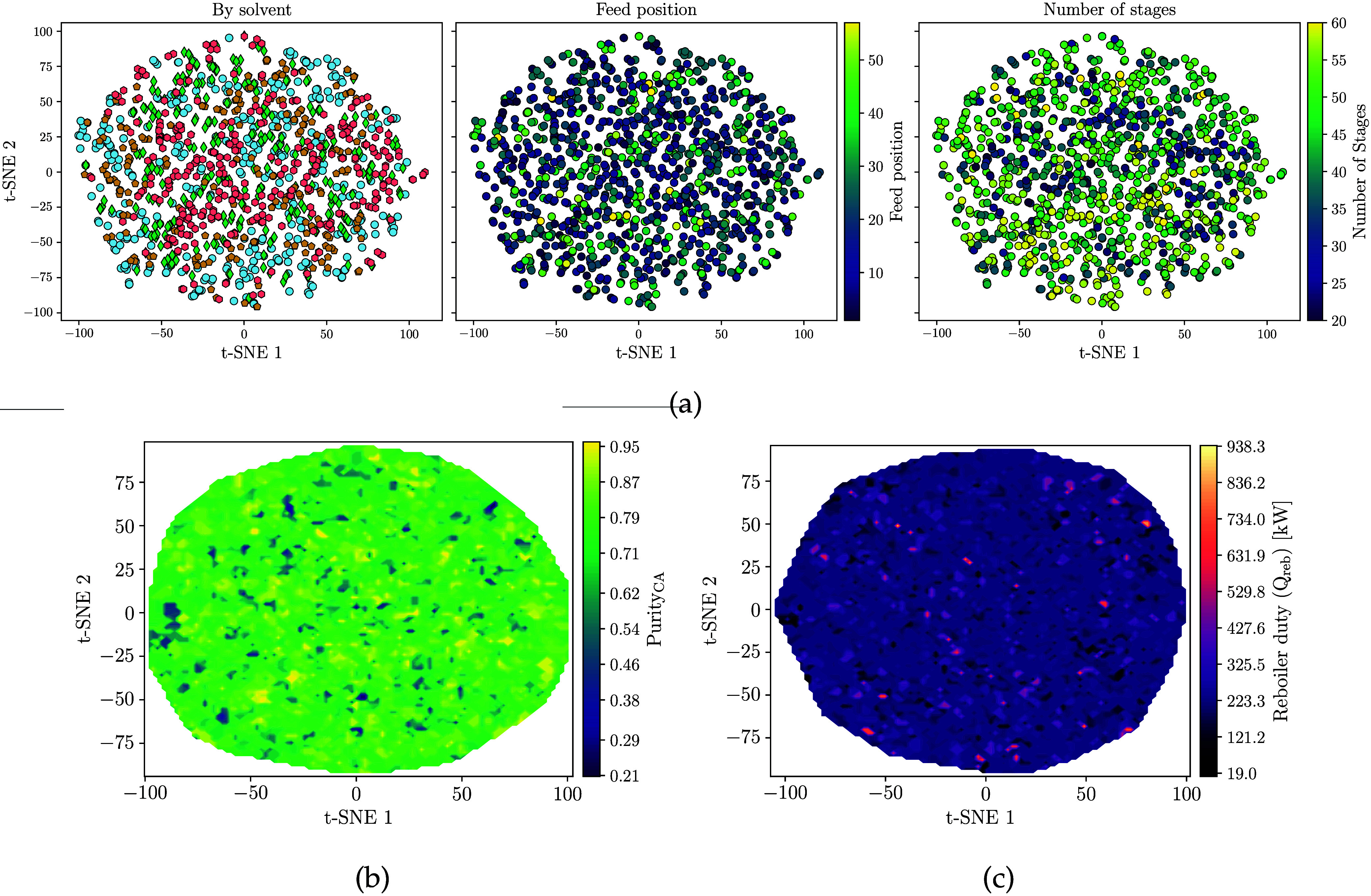
Projection of design in the original space X into latent space Z using reduced with t-SNE; we highlight points corresponding to the solvent used, feeding position, and number of stages (a). Solvents are represented as follows: ○ (blue) oley alcohol, ◊ (green) 1-decanol, ⬠ (brown) 1-octanol, ⎔ (red) *n*-butyl acetate. Visualization of the latent space in terms of values of the objectives of purity (b) and reboiler duty (c).

Figure 7a visualizes the evolution of the BO search over multiple iterations. The GP models were initially trained by using data collected from 10 initial simulations. In the first iteration, we can see that predictions have high uncertainty, which is attributed to the limited amount of data available. We can also see that most points are dominated points (not in the Pareto frontier). As more data are collected, the GPs are refined, allowing for the discovery of more nondominated (Pareto) points and a reduction of uncertainty. For instance, in iteration 30, the uncertainty is significantly reduced along the Pareto frontier, and the predicted frontier shifts toward the bottom right. In iteration 60, we can see that most points begin concentrating on the Pareto frontier (indicated by low uncertainty), while the nondominated region is not visited by BO (indicated by high uncertainty). This indicates that BO is strategically prioritizing the Pareto region.

**Figure 7:**
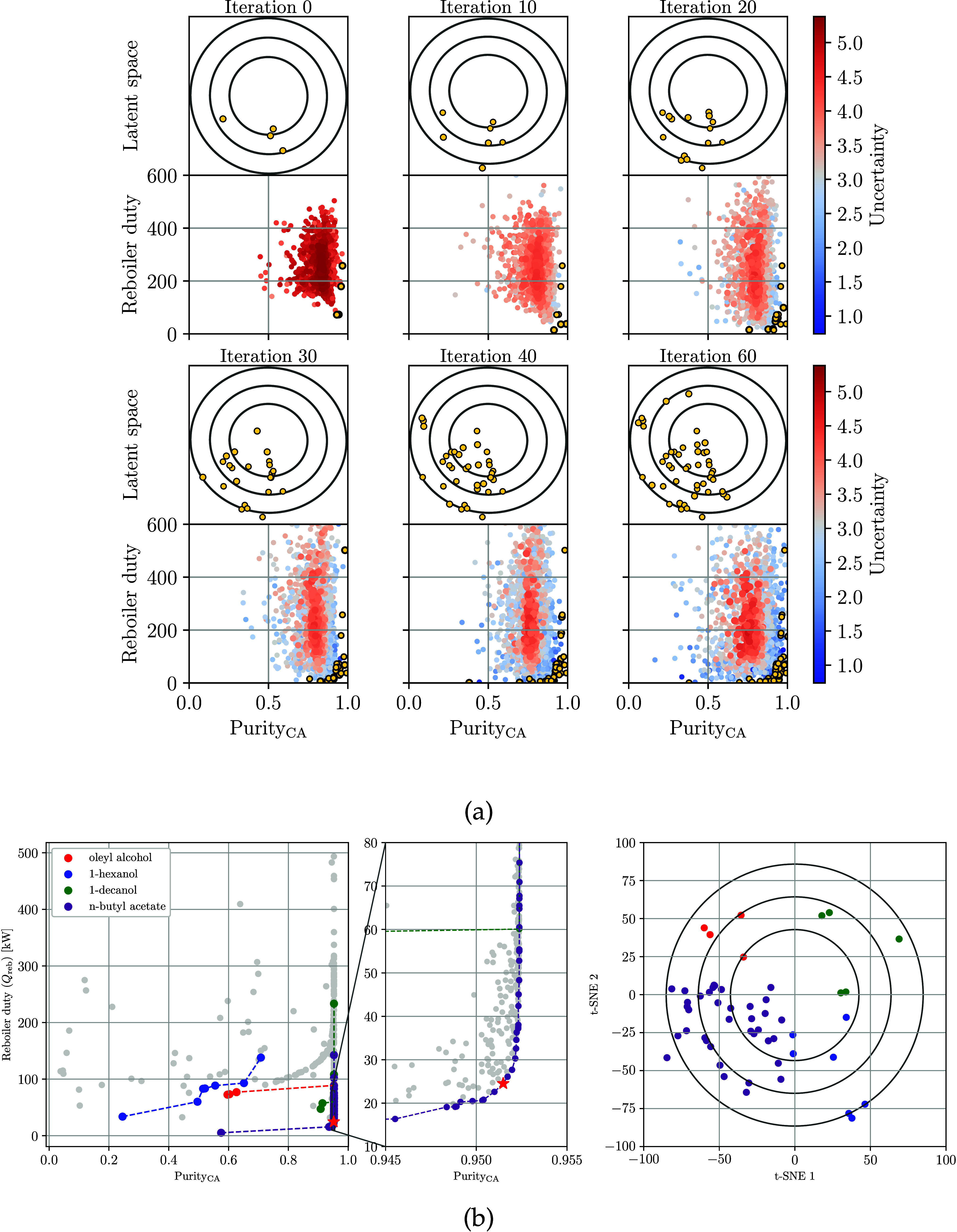
Evolution of the GP models and Pareto frontier along the BO search (a). Yellow dots represent the nondominated (Pareto) points identified by the BO search. The concentric circles are level sets of the latent space, which highlight the positioning of the nondominated points. We can see that the BO search quickly identifies a region in the space that corresponds to nondominated points. Visualization of all simulations obtained with Aspen Plus and visualization of Pareto front highlighting different types of solvents and corresponding locations of the Pareto points in the latent space (b). We can see that the BO search identifies the Pareto frontier and we see that points along the Pareto front correspond to specific region of the latent space.

All results from the simulations conducted with the AspenPlus simulator are shown in Figure 7b. We confirm that, during the search, BO visits regions that are nondominated points (not on the Pareto frontier). It is interesting to observe that different solvents tend to form different Pareto frontiers, but the best frontier is obtained for *n*-butyl acetate. By visualizing the Pareto points of this solvent in the latent space, one can observe that all these points cluster in the same region of the latent space. Here, we can also see that the points corresponding to other solvents are in other regions of the latent space. This highlights that the VAE is constructing a latent space with clearly defined regions.

In Table 2, a summary is given of different designs identified using BO, which enables the assessment of the diversity of the designs and associated objectives, thus providing a clear picture of the solution space explored in the search and the trade-offs involved. The average purity of compound CA (*x*_*CA*_) was obtained as 93.6%, with a relatively wide standard deviation of 8.3%, ranging from a minimum of 50.6% up to a maximum of 95.2%. This indicates that high purities can be achieved, although the system exhibits significant deviations; these deviations are correlated with the necessary amount of solvent required to carry out the separation. The average solvent feed flow rate *F*_*S*_ was 3.11 kmol/h, with a standard deviation of 1.03 kmol/h, which ranged from 1.80 kmol/h to 6.15 kmol/h, reflecting the trade-off between solvent addition and separation performance. We can see that the compromise solution selects *n*-butyl acetate, achieves a purity of 95.14%, and has a reboiler duty of 24 kW.

**Table 2:** Descriptive Statistics of Nondominated Points, Dominated for the Caprylic Acid Recovery Problem and Configuration for the Compromise Solution Found

Point	Metric	*x*_*CA*_ [%]	*Q*_*reb*_ [kW]	*R* [-]	*F*_*S*_ [kmol/h]	*S* ^a^ [-]	*NF*_*stage*_ [-]	*NT*_*stages*_ [-]
Nondominated	mean	93.60	51.0	0.400	3.108	3	24	45
	std	8.3	33.7	0.277	1.032	1	13	11
	min	50.6	10.47	0.110	1.799	2	1	20
	max	95.2	148.29	1.168	6.145	3	57	60
Dominated	mean	88.92	112.9	1.260	3.235	2	23	44
	std	17.44	90.4	0.712	1.506	1	14	11
	min	3.60	16.6	1.112	1.112	0	1	20
	max	95.2	493.6	2.999	9.908	3	59	60
Compromise		95.14	24.54	0.1551	2.25	3	29	44

a Solvents oleyl alcohol, 1-octanol, 1-decanol, and n-butyl acetate are encoded with the categorical values 0, 1, 2, and 3.

For the solvent type (*S*), 1-decanol and n-butyl acetate solvents were found to be the best options in terms of energy requirements. In terms of the reflux ratio (*R*), the average value was 0.40, with a minimum of 0.11 and a maximum of 1.17, indicating that both highly economical and more energy-intensive operating modes were explored. The mean reboiler duty (*Q*_*reb*_) found was 51.0 kW, ranging from 148.2 to 10.5 kW with a standard deviation of 33.7 kW in the most energy-efficient cases. The feed stage number (*NF*_*stage*_) had an average of 24 stages with a standard deviation of 13 stages, extending from a minimum of 1 to a maximum of 57. In contrast, the total number of stages (*NT*_*stages*_) averaged 45, with a minimum of 20 and a maximum of 60. This diversity emphasizes the significant impact of operating conditions on the purity and energy requirements of the distillation process. For the dominated points, the average purity decreases to 88.92, but also provides designs with the maximum recovery. Regarding the reflux ratio, the values of the dominated points have a higher average and reach the upper exploration limit of 2.999. The same behavior can be observed in the case of solvent flow, where the average corresponds to 3.235 kmol/h, with the maximum value explored being 9.908 kmol/h. These dominated points exhibit a wide variety of designs for the different solvents tested with the average provided by 1-decanol as the predominant solvent, which indicates that the BO search is exploring a broad range of designs along the search.

Our findings emphasize the impact of solvent selection on the inherent trade-offs between energy efficiency and column design. The selection of the best solvent can also be justified by comparing the vapor–liquid equilibrium diagrams for each entrainer (see the SI for equilibrium diagrams). From all solvents tested, n-butyl acetate has the lowest boiling point of 463 K; oley alcohol, 1-decanol, and 1-octanol have boiling points of 623, 503, and 468 K, respectively. Nonetheless, the boiling points of n-butyl acetate and 1-octanol are close, making their selection nontrivial and thus requiring an optimization procedure.

The performance curves in SI, Figure S3, show that the proposed MOBO-VAE framework reaches the compromise solution substantially faster than NSGA-II under the same evaluation budget. MOBO-VAE consistently reduces the distance to the compromise point, crossing the 0.5 threshold earlier and maintaining lower distances throughout the optimization. In contrast, NSGA-II converges more slowly and exhibits higher variance. Overall, the results highlight MOBO-VAE’s superior sample efficiency and convergence behavior in the caprylyc acid recovery case study.

### 4.2 Dividing Wall Column

The incorporation of thermally coupled units in chemical processes has generated significant attention due to their benefits by reducing capital and auxiliary utilities costs.^29–31^ Dividing wall columns (DWC), in particular, have been applied in various processes worldwide.^32^ These units have been used for a wide range of applications, including difficult separations (e.g., mixtures with azeotropes). An application we consider here is the separation of organic mixtures generated in the pharmaceutical industry, specifically mixtures of methanol and dichloromethane (DCM). The design of DWCs is challenging due to the tight integration of the system components.

A reported work has focused on identifying designs of DWCs that lead to high purity DCM (≥99.90%).^33^ We extend here such work by evaluating the inherent safety of these columns as part of the optimization problem. The metric used is the Fire and Explosion Damage Index (FEDI), which was proposed by Khan and Abbasi^34^ and has been used for the early risk evaluations.^35,36^ This index was initially proposed for a comprehensive methodology called Hazard Identification and Ranking (HIRA) that was also constituted by a toxicity damage index. Both indices provide a quantitative assessment, but depending on specifics bounds provided by Khan and Abbasi,^34^ a qualitative evaluation can also be obtained. This procedure enables the quantification and classification of risks using either index. In this work, we use the FEDI as an additional objective function due to its computational simplicity. Thus, the formulated problem is defined over the set d∈D , which represents the *design decision variables* listed in Table 3. The goal is to maximize the purity of both distillates while also ensuring the design has optimal safety properties. It is assumed that a lower DCM composition indicates lower methanol purity. Therefore, the purity of DCM (*x*_*DCM*_) is taken as a reference to measure the purity of the other distillate. The problem is stated as follows:

9 $$\begin{array}{cc} \underset{d\in D}{\min} & \left(\mathrm{FEDI}\left(d\right),-x_{DCM}\left(d\right)\right)\\ s.t. & g\left(d\right)\leq 0,\\ & h\left(d\right)=0, \end{array}$$

where **h**(·) = **0** and **g**(·) ≤ **0** represent column model equations and operational design constraints, respectively. The design variables and their corresponding bounds to optimize the DWC are listed in Table 3. A feed flow rate of 100 kmol/h was considered with a couple of possible variations corresponding to equimolar and near-azeotropic concentrations. In the equimolar scenario, both distillate rates are set at 50.05 kmol/h, while the azeotropic feed values are set to 83.07 and 16.97 kmol/h. The thermodynamic equivalent intensified column is shown in Figure 8. For the AspenPlus simulation, the NRTL thermodynamic model was used. The information needed to compute FEDI values and classification ranges is provided in the SI Section 1.6. With the information provided on Table 3, 5000 data points were used to generate the data set $D^{\kappa }$ . Details of the final hyperparameters resulting from the optimization procedure can be found in Table S1. Loss curves for the training and testing sets are provided in Figure S1b.

**Figure 8:**
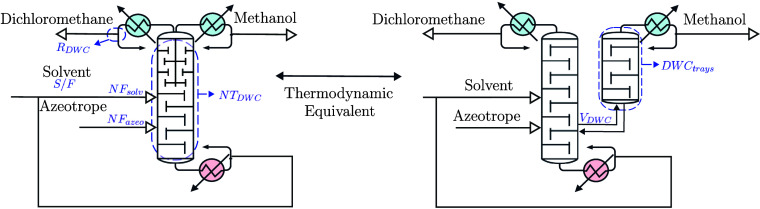
Thermodynamic representation of a dividing wall column for the separation of an azeotropic mixture of dichloromethane and methanol. Blue text indicates all the design variables that are optimized.

**Table 3:** Design Variables to Optimize the DWC and Their Search Ranges

**Design variables**	**Units**	**Range**	**Domain**
Reflux ratio (*R*_*DWC*_)	[-]	(2.0 – 5.0)	Continuous
Vapor flow rate (*V*_*DWC*_)	[kmol/h]	(50 – 100)	Continuous
Solvent to Feed Ratio (*S*/*F*)	[-]	(1.5 – 10.0)	Continuous
Solvent feed stage (*NF*_*solv*_)	[-]	{1 – 50}	Discrete
Azeotrope feed stage (*NF*_*azeo*_)	[-]	{1 – 50}	Discrete
DWC Trays (*DWC*_*trays*_)	[-]	{1 – 50}	Discrete
Total stages (*NT*_*DWC*_)	[-]	{30 – 50}	Discrete

As in the previous case study, we sampled points in the latent space and visualized them using t-SNE. Figure 9 shows this distribution with respect to the discrete variables: solvent and azeotrope feeding position, number of dividing wall stages, and total number of dividing column stages. We can see again that the VAE generates a mixed space that is continuous and follows ellipsoidal regions.

**Figure 9:**
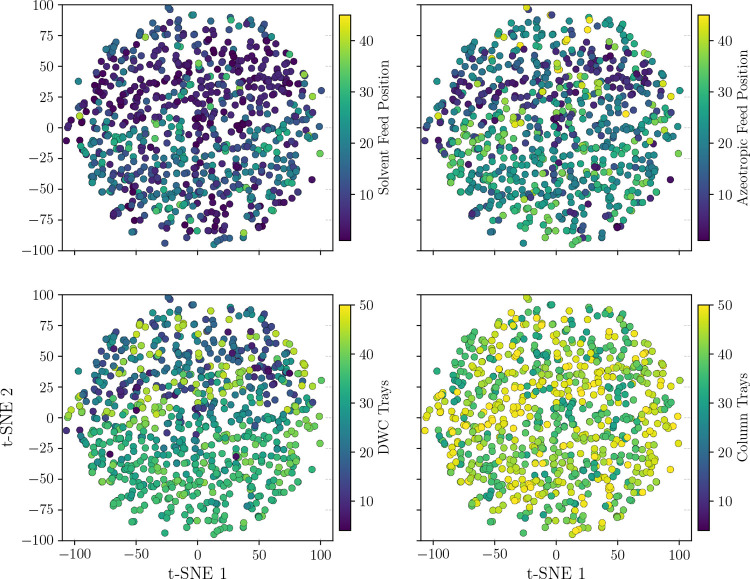
Projection of random sample points of the latent space **z** reduced with t-SNE and their corresponding distribution for the feeding positions of the solvent, azeotrope, size of diving wall, and total column trays.

The evolution of the BO search and their corresponding GP model predictions are shown in Figure 10a. Initially, with 50 data points, the predictions are highly restricted to the range of 0.9–1.0 mole fraction and 325–345 FEDI values. Once new feasible designs are discovered and collected, this space increases mainly in the FEDI region, where high uncertainty is located behind the Pareto front. From iteration 30 onward, it can be seen that the search intensifies within the area of interest. This progressive refinement is based on the scenario where an equimolar composition is assumed. Similarly, it can be seen that the latent points collected throughout the optimization process are distributed well throughout the space; this highlights that there is no particular region in the design space under which the Pareto solutions cluster (which means that this is a complicated search).

**Figure 10:**
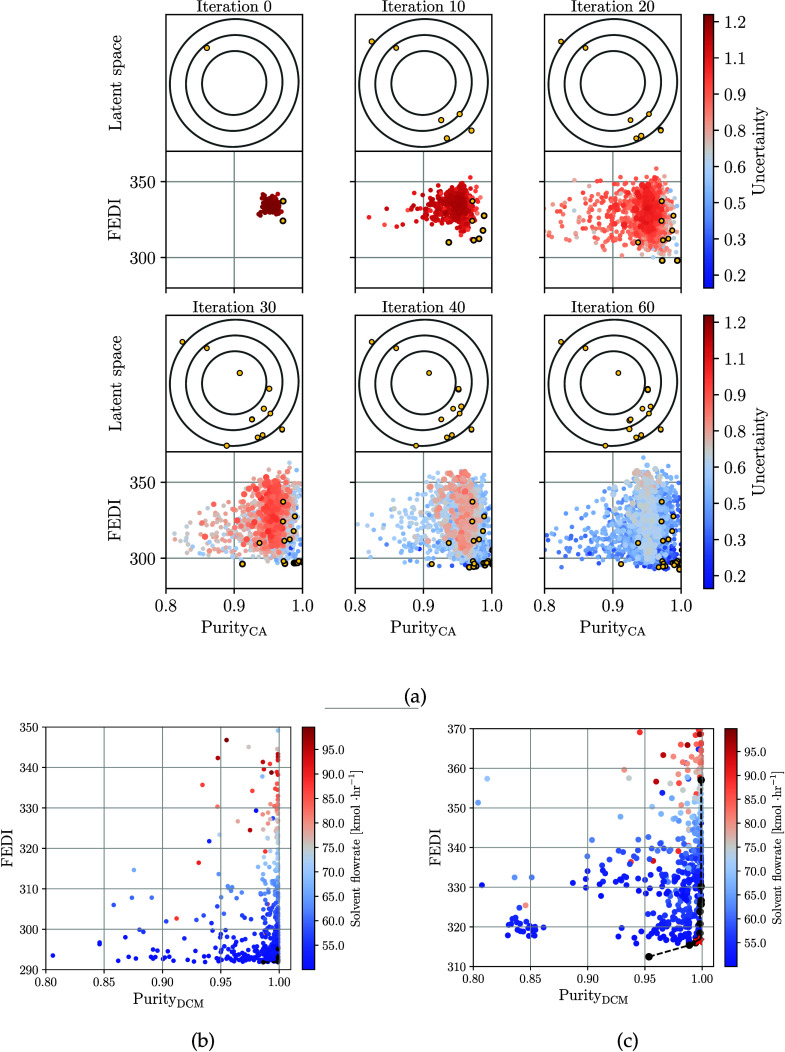
Evolution of BO search thought different iterations with their corresponding GP predictions (a). Yellow dots represent the nondominated (Pareto) points collected. Feasible designs were found for the dividing wall column for equimolar composition (b) and azeotropic composition (c). Points are color-coded according to the solvent flow rate, which is a critical factor for the inherent safety metric. Black dots and dashed lines show the Pareto front.

Figure 10 also illustrates the relationship between composition and the inherent safety FEDI metric. Although the composition of the distillates is relaxed, the intensified unit is still classified as hazardous. In Figure 10b, the index FEDI varies within the range 294–350. However, when the composition of the azeotropic feed changes as shown in Figure 10c, these limits shift to 310–370. These results suggest that the inherent safety index is sensitive to composition perturbations or variations, and that multiple configurations can provide the purity specified, but in some cases, deteriorate the process safety.

The designs identified in Figure 10 were classified into dominated and nondominated solutions, and their corresponding statistics are shown in Table 4. While the results of^33^ correspond to the solution of a mixture with a composition close to the azeotrope, these findings show that optimal solutions considering economy or safety considerations can have conflicts between them. According to their solution, the design that minimized costs operated with a reflux ratio of 0.25 in the main column and incorporated a dividing wall spanning 22 trays, resulting in a total of 31 trays for the DWC. Additionally, the necessary *S*/*F* ratio to meet the purity requirements was 0.48, and the optimal steam split ratio was 0.81. Furthermore, when the objective function changed to the inherent safety, minimizing FEDI reduced this ratio substantially to 0.21 and increased the reflux ratio. Overall, our analysis indicates that the nondominated solutions maintain consistent purity while exhibiting high variations in their safety performance, primarily due to adjustments in the reflux ratio and solvent-to-feed ratio. The dominated points show greater variability with respect to purities and FEDI values, reaching a minimum value of 57.43% and a maximum FEDI of 391.

**Table 4:** Nondominated and Dominated Designs for a Divided Wall Column

	*x*_*DCM*_ [%]	FEDI [-]	*R*_*DWC*_ [-]	*S*/*F* [-]	*V*_*DWC*_ [kmol/h]	*NF*_*solv*_ [-]	*NF*_*azeo*_ [-]	*DWC*_*trays*_ [-]	*NT*_*DWC*_ [-]
Nondominated Points									
mean	99.45	314	2.96	0.56	54.95	7	24	33	43
std	1.08	17	0.81	0.13	10.28	6	4	6	6
min	95.35	291	2.13	0.50	50.11	2	20	26	35
max	99.91	357	4.45	0.97	95.04	22	36	45	50

Dominated Points									
mean	97.25	321	3.47	0.60	54.30	10	21	31	43
std	5.03	23	0.71	0.12	7.32	7	8	8	5
min	57.43	292	2.00	0.50	16.05	1	2	5	30
max	99.91	391	4.99	0.99	99.60	40	43	46	50
c.s^a^	99.91	293.5	4.2	0.50	52.5	4	34	38	42
c.s^b^	99.72	316.1	2.8	0.50	53.1	3	18	31	50

a Optimal solution for an *equimolar* feed.

b Optimal solution for an *azeotropic* feed.

Statistics associated with column dimensionality show that both sets of points explore similar regions, implying that these variables do not have a significant impact on the safety indicator. Therefore, the critical factor identified for these distillation configurations is the amount of solvent used to achieve the separation. From a safety standpoint, minimizing the quantity of flammable materials processed within the unit significantly enhances the overall safety of the unit operation.

Complementary performance results are provided in the SI. Figures S4 and S5 present the performance curves of the proposed MOBO-VAE framework compared to NSGA-II for the extractive dividing-wall column case study with the two different feed compositions. Across both feed conditions, MOBO-VAE demonstrates substantially faster convergence than NSGA-II. For the azeotropic feed case, MOBO-VAE rapidly decreases the distance to the compromise solution and crosses the threshold well before NSGA-II. For the equimolar feed case, the improvement is even more pronounced, with MOBO-VAE not only reaching but surpassing the convergence threshold by several orders of magnitude, maintaining significantly lower distances throughout the optimization.

Overall, the results confirm that MOBO-VAE is markedly more sample-efficient and robust across different feed conditions, consistently outperforming NSGA-II in guiding the search toward high-quality compromise solutions. With the proposed methodology, we effectively navigated and evaluated a complex structure, which can be adapted to enhance more complicated systems, such as reactive dividing wall columns or electrified distillation columns.

## 5 Conclusions and Future Work

This work illustrates how to transform discrete design spaces into continuous one using variational autoencoders (VAEs). We show that this transformation facilitates the implementation of simulation-based optimization approaches, such as Bayesian Optimization (BO). While no strict theoretical guarantee exists that an optimum identified in the latent space corresponds to the global optimum in the original design space, our results show that the VAE provides a latent representation with low reconstruction error. Therefore, the latent space enables effective and reliable exploration, while optimality is always evaluated in the original design space using the high-fidelity process simulator. The proposed framework has been implemented on a CSTR reactor where all decision variables were discretized. This shows the potential applicability to real-world scenarios where experimental campaigns are limited by cost or time, resulting in exploration limited by a fixed budget. In addition, we showed the applicability of the approach to the design of conventional and intensified distillation processes, where the numerical convergence and the number of evaluations represent a computational limitation. In this sense, this approach can be applied to more complex distillation columns, such as multiproduct dividing wall columns and thermally coupled reactive columns, where exploration costs are even higher. We show that the proposed approach is effective at navigating the design space and can quickly discover optimal solutions. The performance of the proposed approach has been evaluated via computational experiments.

As part of future work, we would like to conduct additional studies to determine the ability of transforming different types of design spaces and to provide a more rigorous justification of performance. We are also interested in exploring the ability of the VAE approach to represent discrete spaces that have complex geometries (as identified by constraints) and we are interested in using the design space transformation approach to other types of problems, such as purely continuous optimization problems. On the other hand, a critical challenge of this methodology lies in the structure of the VAE, such as the architecture of the encoder and decoder, and the hyperparameters used. Improvement of the hyperparameter is essential to reduce reconstruction loss, avoiding unfeasible reconstructed points. Specifically, the embedding-layer dimension for each variable is assumed to be the same across all discrete variables, although it could be optimized for each variable. In addition, the dimensionality of the latent space plays a crucial role in determining the overall performance of the VAE. While it provides a compact representation of the underlying data, an excessively high or low latent dimension can limit the model’s expressiveness and generalization ability. To mitigate this constraint, the search space can be adaptively partitioned through Bayesian Optimization techniques combined with trust-region methods, thereby accelerating the discovery of optimal solutions and enhancing the construction of a well-defined Pareto front.

## Supplementary Material



## Notes

Sections 1.1 and 1.2: Information related to design and performance on VAE is provided; Section 1.2: Standard algorithm for continuous variables with BO; Section 1.3: Information related to design and performance on VAE is provided; Section 1.4: Thermodynamic data of the NRTL is added to reproduce results of the caprylic acid recovery case; Section 1.5: Vapor-Liquid Equilibrium Diagrams; Section 1.6: FEDI equations required to calculate it are included; Section 1.7: MOBO-VAE and NSGA-II performance (PDF)
