# Supplementary material for: Simulation-Based Optimization over Discrete Spaces Using Projection to Continuous Latent Spaces
Source: Ind Eng Chem Res. 2026 Feb 13;65(7):3999–4012. doi: 10.1021/acs.iecr.5c04315 (PMC12947695; doi:10.1021/acs.iecr.5c04315)
Supplement: Supplementary file 1 [file ie5c04315_si_001.pdf]

Simulation-based Optimization over Discrete Spaces using Projection to Continuous Latent Spaces

Gabriel Hernández-Morales[†,‡], Brenda Cansino-Loeza[†]
Arturo Jiménez-Gutiérrez[‡], Victor M. Zavala[†*]

[†]Department of Chemical and Biological Engineering
University of Wisconsin - Madison, 1415 Engineering Dr, Madison, WI 53706, USA
[‡] Department of Chemical Engineering
Tecnologico Nacional de Mexico - Instituto Tecnologico de Celaya, Celaya, Guanajuato, Mexico

1 Supplementary data

1.1 Hyperparameters

The optimal hyperparameters found for each case study are shown in Table S1. The Optuna package was used to find the optimal parameters using 50 trials [1].

Table S1: Optimal values found for the hyperparameters of the VAE for each case study.

	**Toy Example**	**Case 1**	**Case 2**
Embbeding dimension	(16,16,4)	(8,8,8)	(71, 71, 71, 71)
Encoder dimensions	(36,64)	(24,16)	(284,115, 57,28)
Decoder dimensions	(64,36)	(16,24)	(28,57,115,284)
LeakyReLU slope	0	0.1	0.01
Learning rate α	$1e^{-2}$	$1.553e^{-4}$	$2.222e^{-4}$
Weighting parameter β	0.0001	0.1027	0.0517
z dimension	8	8	7

1.2 VAE Losses

This section presents the training and test losses for each VAE used in the case studies. These plots are obtained by setting as optimal hyperparameters those obtained in Table S1. The time needed to train the VAE for caprylyc recovery and the extractive distillation was 48 min and 15

*Corresponding Author: victor.zavala@wisc.edu.

min, respectively. VAE training was performed on a personal workstation with an Intel® Core™ i7-8750H CPU (2.20 GHz) and 32 GB RAM.

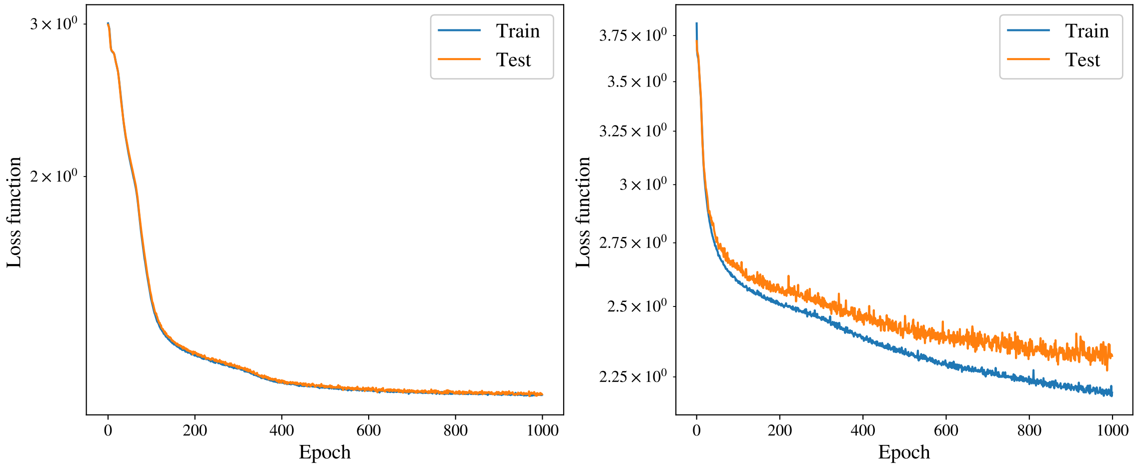

(a) (b)

Figure S1: Training and testing curve for the VAEs trained for the caprylic acid recovery S1a and the extractive dividing wall column S1b.

1.3 Bayesian Optimization

In this section, we describe the generalized methodology for implementing Bayesian Optimization, where no reformulation is needed since the initial data are within the continuous domain. First, a Gaussian Process ($\mathcal{GP}$) provides a posterior distribution over function values given observed data $x \in \mathbb{R}_+$ that is assumed to be continuous. The GP is defined by a mean function $m(x) \in \mathbb{R}^d$ and a covariance function $\mathbf{K}(x, x^{'})$, such that $f(x) \sim \mathcal{N}(m(x), \mathbf{K}(x, x^{'}))$. The $\mathbf{K}(x, x^{'})$ can be calculated from a wide variety of kernel functions, such as the Radial Basis Function (RBF), the Matern, and Rational Quadratic (RQ) kernels. The Matern kernel generalizes the RBF by including a parameter v to control the smoothness of the generated function, and it is commonly used to construct the covariance matrix.

$$\mathbf{K}(x, x') = \sigma^2 \frac{2^{1-\nu}}{\Gamma(\nu)} \left(\frac{\sqrt{2\nu}\, \|x - x'\|}{l} \right)^{\nu} K_{\nu} \left(\frac{\sqrt{2\nu}\, \|x - x'\|}{l} \right) \tag{1}$$

where σ^2 is the process variance, l is the scale length, $\nu > 0$ controls the smoothness, $\Gamma(\cdot)$ is the Gamma function, $K_\nu(\cdot)$ is the modified Bessel function of the second kind, and $\|x - x'\|$ is the Euclidean distance. The GP constructs a predictive posterior distribution over the function value at a new experimental point $\mathbf{x}$, providing a Gaussian approximation $\hat{f}^{\ell}(x) \sim \mathcal{N}(\mu_f^{\ell}(x), \sigma_f^{\ell}(x))$ of the true function $f(x)$. The predicted mean and variance are given by

$$\mu_f^{\ell}(x) = \mathbf{K}(x, x_{\mathcal{K}}^{\ell})\mathbf{K}(x_{\mathcal{K}}^{\ell}, x_{\mathcal{K}}^{\ell})^{-1} f_{\mathcal{K}}^{\ell} \tag{2}$$

$$\sigma_f^\ell(x) = \mathbf{K}(x,x) - \mathbf{K}(x,x_{\mathcal{K}}^\ell)^T \mathbf{K}(x_{\mathcal{K}}^\ell, x_{\mathcal{K}}^\ell)^{-1} \mathbf{K}(x_{\mathcal{K}}^\ell, x_{\mathcal{K}}) \tag{3}$$

The kernel hyperparameters (lengthscales, signal variance, and noise variance) are learned by maximizing the exact marginal log-likelihood, while the Matérn smoothness parameter ν is fixed to 2.5. In Bayesian Optimization (BO), the Gaussian Process (GP) surrogate model is used not only to approximate the expensive objective function but also to guide the selection of the next evaluation point through an acquisition function. Acquisition functions (AF) balance the trade-off between exploration (sampling in regions of high uncertainty) and exploitation (sampling in regions where the surrogate predicts low function values). By maximizing the acquisition function, BO sequentially proposes candidate points that are likely to improve upon the current best solution.

One of the most widely used acquisition functions is the Expected Improvement (EI). The EI criterion quantifies the expected improvement of a new sample x over the current best observation f^+, given the predictive posterior distribution of the GP. It is defined as

$$\mathrm{EI}(x) = \mathbb{E}\big[\max(0, f^+ - f(x))\big]\,, \tag{4}$$

which can be expressed in closed form as

$$\mathrm{EI}(x) = (f^+ - \mu_f(x) - \xi)\,\Phi\left(\frac{f^+ - \mu_f(x)}{\sigma_f(x)}\right) + \sigma_f(x)\,\phi\left(\frac{f^+ - \mu_f(x)}{\sigma_f(x)}\right), \tag{5}$$

where $\mu_f(x)$ and $\sigma_f(x)$ are the predicted mean and standard deviation of the GP at point x, f^+ is the best function value observed so far, $\Phi(\cdot)$ is the cumulative distribution function, and $\phi(\cdot)$ is the corresponding probability density function (PDF). ξ, a hyperparameter that defines exploration and exploitation, is fixed to zero. The EI acquisition function thus favors points with both high predicted mean improvement (exploitation) and high predictive uncertainty (exploration), making it a robust choice in practice. Once x is sampled by evaluating AF, the data is collected and the process is performed n times until a limit budget is reached.

1.4 Thermodynamic data

In this section are listed all the thermodynamic data required to reproduce the simulations in each case study. This information is collected from the simulation software Aspen and the data corresponds to a non-ideal thermodynamic model, the NRTL. As all mixtures exhibited high deviations from ideal state, we use this data to predicted high non-linearities within the system.

Table S2: Thermodynamic data used for the NRTL model for the case study of the recovery of caprylic acid.

Component i	Component j	A_{ij}	A_{ji}	B_{ij}	B_{ji}	C_{ij}
Caprylic acid	Water	0	0	179.748239	2040.78591	0.3
Caprylic acid	oleyl alcohol	0	0	641.193769	-450.023621	0.3
Water	oleyl alcohol	0	0	5151.32066	480.615554	0.3
Caprylic acid	1-decanol	0	0	513.04533	-382.731915	0.3
Water	1-decanol	9.1803	-0.1658	277.5351	150.2704	0.2
Caprylic acid	1-octanol	0	0	450.707838	-346.171072	0.3
Water	1-octanol	5.9173	-1.4468	798.3772	741.9184	0.26
Caprylic acid	n-butanol	0	0	-277.519581	532.346082	0.3
Water	n-butanol	6.5745	1.4822	-174.5521	203.3306	0.3

Table S3: Thermodynamic data used for the NRTL model for the azeotropic methanol-dichloromethane case study using dimethylformamide as solvent.

Component i	Component j	A_{ij}	A_{ji}	B_{ij}	B_{ji}	C_{ij}
Methanol	Dichloromethane	0	0	-102.5306	812.7825	0.3
Methanol	Dimethylformamide	-0.501	-0.8325	712.2657	-208.2091	0.3
Dichloromethane	Dimethylformamide	-2.74351	-9.448972	1401.151	3263.881	0.3

1.5 Vapor-Liquid Equilibrium Diagrams

In Figure S2 are shown the vapor and liquid equilibrium diagrams for each solvent selected for the recovery of caprylic acid.

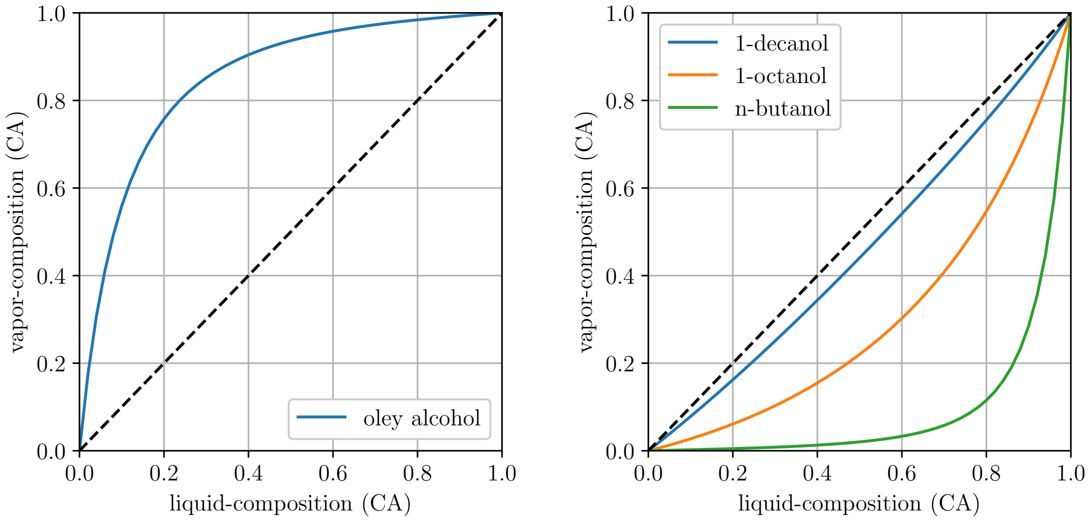

(a) (b)

Figure S2: Figure S2a shows the vapor-liquid diagram for the oley alcohol solvent. Figure S2b illustrates the diagrams corresponding to 1-decanol, 1-octanol, and n-butyl acetate.

1.6 Fire & Explosion Damage Index

This section describes in detail the equations required to calculate the Fire & Explosion Damage Index (FEDI), which is used to assess the inherent safety of dividing wall columns. The index is determined based on four factors: chemical energy (as represented in Equation 6), physical energy (detailed in Equations 7 and 8), and the energy associated with the operation of chemical reactors (shown in Equation 9).

$$F_1 = 0.1(M)(\frac{H_c}{K}) \tag{6}$$

$$F_2 = 1.30 \cdot 10^{-3}(PP)(Vol) \tag{7}$$

$$F_3 = 1 \cdot 10^{-3}(\frac{1}{T + 273.15})(PP - VP)^2(Vol) \tag{8}$$

$$F_4 = (M)(\frac{H_{rxn}}{K}) \tag{9}$$

In these equations, M represents the mass flow rate in kg/s, while H_c and H_{rnx} indicate the enthalpy of combustion and reaction, respectively, measured in kJ/kg. The constant K is set to 3.148. Additionally, T denotes the temperature in Kelvin (K), VP stands for the vapor pressure in

kilopascals (kPa), PP refers to the operating pressure in kilopascals (kPa), and Vol indicates the volume of the equipment in cubic meters (m^3). Penalty functions are implemented to estimate a $hazard\ \ potential$ depending on the classification of each unit involved within the process.

$$hazard\ \ potential_{Group_I} = [(F1_{FEDI})(pn_1) + (F)(pn_2)] \left(\prod_{i=3}^{8} pn_i \right) \tag{10}$$

$$hazard\ \ potential_{Group_{II}} = [(F1_{FEDI})(pn_1) + (F)(pn_2)] \left(\prod_{i=3}^{8} pn_i \right) \tag{11}$$

$$hazard\ \ potential_{Group_{III}} = [(F1_{FEDI})(pn_1) + (F)(pn_2) + (F4_{FEDI})(pn_9)(pn_{10})] \left(\prod_{i=3}^{8} pn_i \right) \tag{12}$$

$$hazard\ \ potential_{Group_{IV}} = [(F1_{FEDI})(pn_1) + (F)(pn_2)] \left(\prod_{i=3}^{9} pn_i \right) \tag{13}$$

$$hazard\ \ potential_{Group_V} = (F1_{FEDI}) \left(\prod_{i=1}^{8} pn_i \right) \tag{14}$$

Finally, the $hazard\ \ potential$ is used to calculate the FEDI as shown in Equation 15. The FEDI results can be ranked based on the values shown in Table S4. Ultimately, the FEDI value indicates the damage radius in meters.

$$FEDI = 4.76 \cdot \sqrt[3]{hazard\ \ potential} \tag{15}$$

Table S4: Lower and upper bounds for the hazard classification based on the FEDI index [2]

**Lower bound**	**Upper bound**	**Hazard classification**
500	$>$	Extremely hazardous
400	500	Highly hazardous
200	400	Hazardous
100	200	Moderately hazardous
20	100	Less hazardous
$<$	20	No hazardous

1.7 MOBO-VAE and NSGA-II perfomance

This sections shows the overall perfomance between the MOBO-VAE framework and the NSGA-II perfomance.

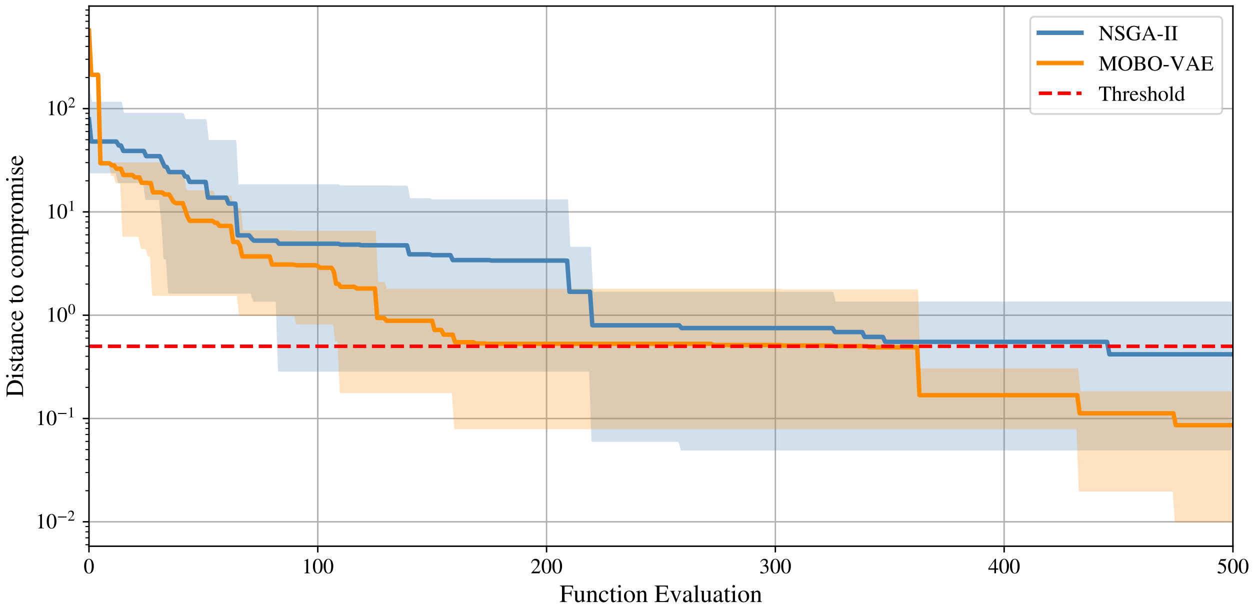

Figure S3: Performance curve of MOBO-VAE and NSGA-II for the caprylyc acid recovery case study. The red dashed line indicates a Euclidean distance of 0.5, indicating that the algorithm is converging toward the compromise solution.

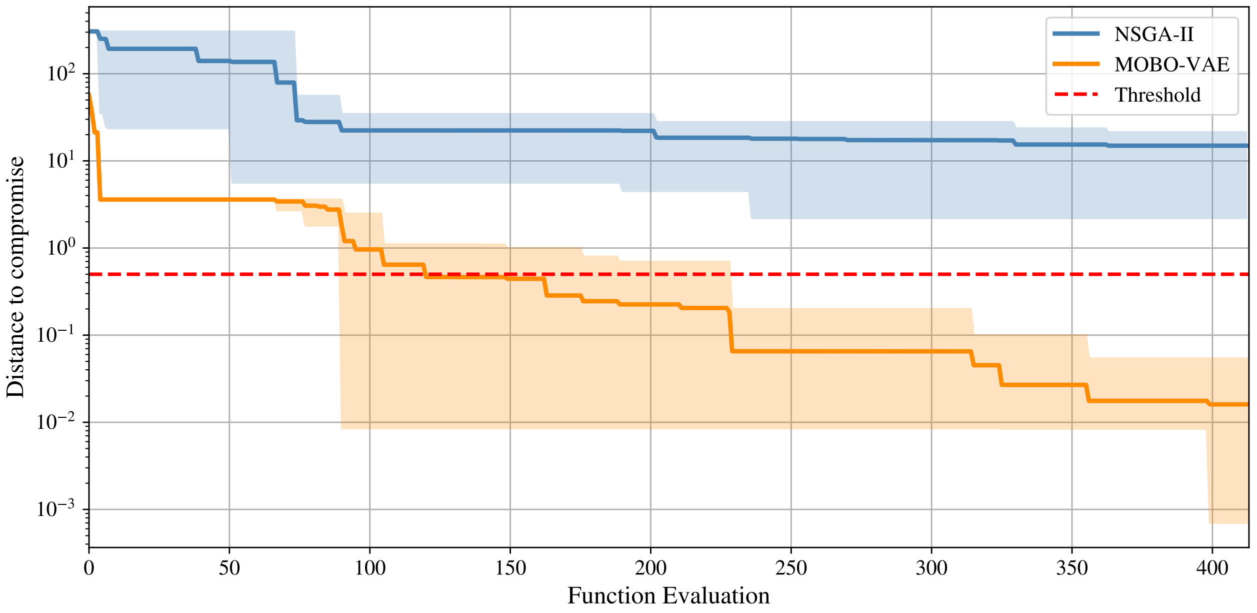

Figure S4: Performance curve of MOBO-VAE and NSGA-II for the extractive dividing wall case study considering an azeotropic feed. The red dashed line indicates a Euclidean distance of 0.5, indicating that the algorithm is converging toward the compromise solution.

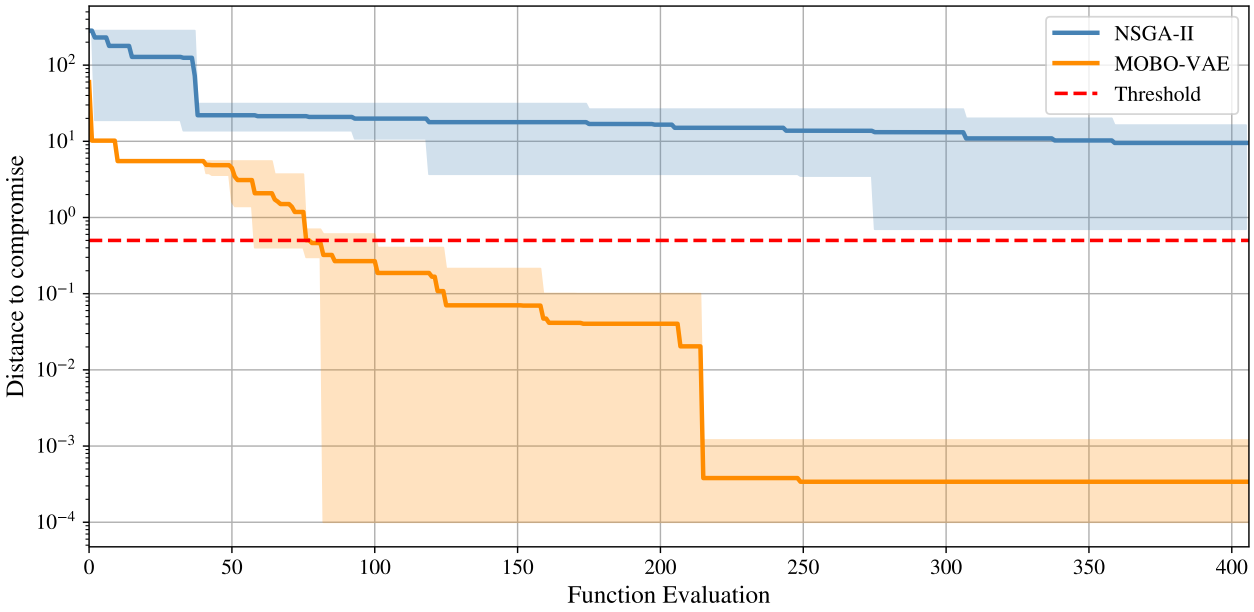

Figure S5: Performance curve of MOBO-VAE and NSGA-II for the extractive dividing wall case study considering an equimolar feed. The red dashed line indicates a Euclidean distance of 0.5, indicating that the algorithm is converging toward the compromise solution.

References

[1] Takuya Akiba, Shotaro Sano, Toshihiko Yanase, Takeru Ohta, and Masanori Koyama. Optuna: A next-generation hyperparameter optimization framework. In *Proceedings of the 25th ACM SIGKDD International Conference on Knowledge Discovery and Data Mining*, 2019.

[2] Faisal I. Khan and S. A. Abbasi. Multivariate hazard identification and ranking system. *Process Safety Progress*, 17(3):157–170, 1998.
